# Internal Structure of Thermoresponsive Physically Crosslinked Nanogel of Poly[*N*-(2-hydroxypropyl)methacrylamide]-*Block*-Poly[*N*-(2,2-difluoroethyl)acrylamide], Prominent ^19^F MRI Tracer

**DOI:** 10.3390/nano10112231

**Published:** 2020-11-10

**Authors:** David Babuka, Kristyna Kolouchova, Ondrej Groborz, Zdenek Tosner, Alexander Zhigunov, Petr Stepanek, Martin Hruby

**Affiliations:** 1 Institute of Macromolecular Chemistry, Czech Academy of Sciences, Heyrovského náměstí 2, 162 06 Prague 6, Czech Republic; babuka@imc.cas.cz (D.B.); kolouchova@imc.cas.cz (K.K.); ondrej.groborz@gmail.com (O.G.); zhigunov@imc.cas.cz (A.Z.); stepanek@imc.cas.cz (P.S.); 2 Institute of Physics, Faculty of Mathematics and Physics, Charles University in Prague, Ke Karlovu 3, 121 16 Prague 2, Czech Republic; 3 Department of Physical and Macromolecular Chemistry, Faculty of Science, Charles University, Hlavova 8, 128 43 Prague 2, Czech Republic; 4 Department of Organic Chemistry, Faculty of Science, Charles University, Hlavova 8, 128 43 Prague 2, Czech Republic; 5 Institute of Biophysics and Informatics, Charles University, First Faculty of Medicine, Salmovská 1, 120 00 Prague 2, Czech Republic; 6 Department of NMR Spectroscopy, Faculty of Science, Charles University, Hlavova 8, 128 43 Prague 2, Czech Republic; zdenek.tosner@natur.cuni.cz

**Keywords:** fluorine-19, magnetic resonance imaging, self-assembly, diblock copolymer, PHPMA, PDFEA

## Abstract

Fluorine-19 MRI is a promising noninvasive diagnostic method. However, the absence of a nontoxic fluorine-19 MRI tracer that does not suffer from poor biodistribution as a result of its strong fluorophilicity is a constant hurdle in the widespread applicability of this otherwise versatile diagnostic technique. The poly[*N*-(2-hydroxypropyl)methacrylamide]-*block*-poly[*N*-(2,2-difluoroethyl)acrylamide] thermoresponsive copolymer was proposed as an alternative fluorine-19 MRI tracer capable of overcoming such shortcomings. In this paper, the internal structure of self-assembled particles of this copolymer was investigated by various methods including 1D and 2D NMR, dynamic light scattering (DLS), small-angle X-ray scattering (SAXS) and small-angle neutron scattering (SANS). The elucidated structure appears to be that of a nanogel with greatly swollen hydrophilic chains and tightly packed thermoresponsive chains forming a network within the nanogel particles, which become more hydrophobic with increasing temperature. Its capacity to provide a measurable fluorine-19 NMR signal in its aggregated state at human body temperature was also investigated and confirmed. This capacity stems from the different fluorine-19 nuclei relaxation properties compared to those of hydrogen-1 nuclei.

## 1. Introduction

Magnetic resonance imaging (MRI) is a well-established non-invasive diagnostic method. The most widely used proton ^1^H MRI has found a vast range of applications clinically and in research, but certain anatomical and/or pathological structures are hard or impossible to visualise [1,2,3]. To overcome this problem, a *T*_1_ (or less commonly *T*_2_) contrast agent (CA) can be employed as they accumulate in certain tissues and alter their relaxation properties, thus enabling their visualisation. Unfortunately, many of these CAs contain toxic paramagnetic metal ions such as Gd^3+^, which limits their applicability [4,5].

Another relatively unexplored approach to widen the potential of MRI is the detection of nuclei other than ^1^H protons. One of the most promising nuclides is the ^19^F isotope, since the ^19^F MRI has very little background signal in human body tissue, high sensitivity (≈83% of ^1^H) and its compounds exhibit a wide range of chemical shifts. Furthermore, fluorinated tracers for ^19^F MRI can be easily designed to be nontoxic and biocompatible. One of their great advantages is their ability to measure ^19^F MRI in commonly used ^1^H MRI devices [2,6,7,8,9]. Nevertheless, ^19^F MRI small-molecule tracers suffer from strong fluorophilicity, leading to excessive hydrophobicity, which often causes an unfavourable biodistribution in the human body. This problem can be overcome by using macromolecular fluorinated tracers, whose biodistribution can be tailored to the required application by varying the polymer composition and architecture [3,10,11,12,13].

Nanoparticles have emerged as highly important materials in medicine and have wide scale clinical applications. In general, nanoparticles show great promise in medical applications both as therapeutic and diagnostic tools, either in combination with other drugs (as a drug delivery system) or on their own if they are bioactive [14,15]. Vasculature of some tumours promotes the permeation and retention of nanoparticles smaller than 200 nm into the tumour tissue. This enhanced permeation and retention (EPR) effect can be used to deliver higher therapeutic or diagnostic doses into the tumour tissue [16]. Currently, a variety of nano-sized systems have been used in biomedical applications, such as quantum dots (fluorescence imaging of lymph nodes [17], lung blood vessels [18], tumour tissue [19]), superparamagnetic iron oxide particles (MRI contrast agents for imaging of tumour tissue [20]) and polymer and liposome-based drug delivery systems (cancer therapy [21,22,23], HIV therapy [24], ocular disease therapy [25], respiratory disease therapy [26], neurodegenerative therapy [27,28]). An interesting area of polymer nanoparticle research is the development of stimuli-responsive nanoscale polymer particles that are capable of self-assembly/disassembly with external stimuli (e.g., temperature) [29,30,31]. Most of these systems are based on thermoresponsive poly(*N*-isopropylacrylamide), with a lower critical solution temperature of approximately 32 °C; in combination with hydrophilic polymers as diblock copolymers, these systems are capable of creating polymer nano-sized assemblies at body temperature with several clinical applications as drug delivery systems [32]. Their success in this regard is based on several factors. The first advantage is that, due to their size, they are not promptly expelled from the human body via renal filtration as the assembled systems are larger than the limit for renal excretion [33]. Another advantage is that they can be expelled from the human body over time as the particles disassemble to unimers, which are expelled through renal filtration, so they are physically biodegradable and do not accumulate in the human body. Finally, polymer systems can be tailored to a specific application and designed, for example, to be invisible to the immune system, so they will not provoke any inflammatory or allergic reactions [33,34,35].

In our previous research, we described self-assembled nanoparticles with similar properties, which were based on thermoresponsive diblock copolymers. These copolymers were composed of a hydrophilic poly[*N*-(2-hydroxypropyl)methacrylamide] (PHPMA) or poly(2-methyl-2-oxazoline) (PMOX) block and a thermoresponsive poly[*N*-(2,2-difluoroethyl)acrylamide] (PDFEA) block [3,10,12]. PHPMA is well known for its excellent biocompatibility and ability to hide nanoparticles from the immune system (the “stealth” effect) [36], and the PMOX shows similar if not better properties in this regard [37]. The hydrophilic blocks sterically stabilize the system in aqueous media and protect the thermoresponsive fluorinated core against unwanted nonspecific interaction with the biological environment. The PDFEA polymer has thermoresponsive properties in that its hydrophobicity increases with temperature [38,39]. By combining these two types of polymers, we obtained systems capable of self-assembly at human body temperature. The obtained nanoparticles were shown to provide a good signal for ^19^F NMR and MRI [10]. As there is very little fluorine in the human body (i.e., the fluorine background signal in human tissue is low), these particles could potentially be used for the imaging of solid tumours due to the above-mentioned EPR effect. However, the question of the origin of the fluorine signal in condensed particles was not considered in the previous research [3].

The internal structure of self-assembled polymer particles is an important factor that influences the ability to carry drugs as well as their biodistribution [12]. In our case, the internal structure of PHPMA-*b*-PDFEA copolymer particles could also be the cause of their ability to provide a significant ^19^F MRI signal. To test this hypothesis, we present a detailed investigation of their internal structure using a wide range of physical methods such as dynamic light scattering, small-angle X-ray scattering, small-angle neutron scattering, ^1^H and ^19^F NMR, Diffusion-Ordered NMR Spectroscopy (DOSY) and Nuclear Overhauser Effect Spectroscopy (NOESY), as we proved in our previous research [12] that this combination of methods can lead to a greater understanding of the internal structure of certain types of nanoparticles, such as nanogels, and can have wider applicability to improve the understanding of researched copolymer nanoparticles.

## 2. Materials and Methods

### 2.1. Materials

**Scheme 1:**
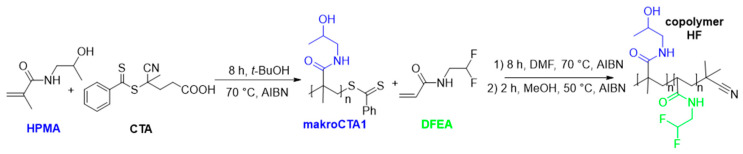
Synthesis of diblock poly[*N*-(2-hydroxypropyl)methacrylamide]-*block*-poly[*N*-(2,2-difluoroethyl)acrylamide] polymer (HF).

D_2_O (99.98%) was purchased from Eurisotop (Saint-Aubin, France). Phosphate-buffered saline (PBS) and the remaining chemicals were purchased from Sigma-Aldrich s.r.o. (Prague, Czech Republic). A block copolymer poly[*N*-(2-hydroxypropyl)methacrylamide]-*block*-poly[*N*-(2,2-difluoroethyl)acrylamide] in a 1:2 ratio (denoted further as HF) was prepared by two sequential reversible addition-fragmentation chain-transfer (RAFT) polymerizations (Scheme 1) using 4-cyano-4-(phenylcarbonothioylthio)pentanoic acid as the chain transfer agent (CTA) and 2,2′-azobis(2-methylpropionitrile) (AIBN) as the initiator. The CTA end-group was removed after polymerization by radical reaction with AIBN. The molar masses of polymer blocks PHPMA and PDFEA were 13.3 and 38.2 kDa, respectively, resulting in a total molar mass of the copolymer of 51.5 kDa [10]. The dispersity of the copolymer (*Đ* = *M*_w_/*M*_n_) has been determined to be 1.07 by Size Exclusion Chromatography (SEC, Figure S1), and the purity and the block ratios in the copolymers were determined by ^1^H NMR (Figure S2) using the methylene protons of the CHF_2_ group of PDFEA (δ = 5.9) and the proton coupled with the carbon atom of the CHOH group of PHPMA (δ = 3.9).

### 2.2. Dynamic Light Scattering (DLS)

In order to investigate the thermoresponsive behaviour of our system in different aqueous solutions (in 150 mM PBS, H_2_O and D_2_O with a polymer concentration of 1 mg∙mL^−1^), we measured the dynamic light scattering using the Zetasizer Nano series Nano-ZS instrument, Model ZEN3600 (Malvern Instruments, UK) in a temperature range of 10 to 50 °C and with a scattering angle of *θ* = 173°. The semi-micro cuvettes were used with a low volume of solution to provide faster thermal equilibration and lower the sample size required. Before the measurement, the sample was left to stabilize at 10 °C for 25 min. Afterwards, the temperature was increased up to 50 °C with a step of 1 °C (equilibration for 5 min for each temperature step). After the measurement, the sample was cooled to 10 °C in one step, and the DLS data were collected in order to analyse the reversibility and hysteresis of the self-assembly system. The measured data were then processed using a series of programmes (Zetasizer software v.7.11, Malvern Instruments Ltd., UK; Excel, Microsoft Office Standard 2019, v.1808, Microsoft Corporation, USA; Matlab v.9.9.0.1467703 (R2020b), The MathWorks, Inc., USA; JMalgen v.2.0, Institute of Macromolecular Chemistry CAS, Czech Republic; and Genr v.11, Institute of Macromolecular Chemistry CAS, Czech Republic [40]) to either transform data files for compatibility purposes, analyse the raw data or compile the processed data into a readable output.

The intensity-based size distributions (A(*R*_h_)) with equal-area representation are presented in our work as those are the only ones that represent the sample as it was measured [41]. This means that because larger particles scatter a greater quantity of light than smaller particles—as the intensity of scattered light is directly proportional to the third power of hydrodynamic radius of a particle (~*R*_h_^3^)—their contribution to the scattered light is much larger than that which would correspond to the pure mass of polymer contained in the larger particles. We do not use recalculation to volume or number-based size distributions, as without the precise knowledge of the form factor of the particles present, these recalculations could easily introduce major errors into the obtained size distributions.

### 2.3. Small-Angle X-ray Scattering (SAXS)

The SAXS data were collected using a pinhole camera (MolMet, Rigaku, Japan, modified by SAXSLAB/Xenocs) on a microfocused X-ray beam generator (Rigaku MicroMax 003) operating at 50 kV and 0.6 mA (30 W). Vacuum version of a Pilatus 300 K detector was used with the camera. Primary beam position and sample-to-detector distances were calibrated using an AgBehenate sample. Borosilicate capillaries were used as measurement containers for the samples. To cover the range of scattering vectors (*q*) from 0.005 to 1.100 Å^−1^, two experimental settings were used.

(1) $$q=\frac{4\pi }{\lambda }\sin\theta$$

where 2θ is the scattering angle, and λ is the incident wavelength.

We chose 22 °C and 40 °C as the measurement temperatures to obtain data throughout the thermally induced self-assembly process. Obtained scattering data were reduced using software based on the PyFAI Python library [42], and SASFit [43] and SASView [44] were used to fit the data.

### 2.4. Small-Angle Neutron Scattering (SANS)

The SANS experiments were performed at the Rutherford Appleton Laboratory, ISIS, UK, using the SANS2d instrument. Neutron wavelengths in the range of 2.0 to 14.0 Å were used. Two detector distances, 4 m and 12 m, were used and thus, a total merged *q* in range from 0.00156 to 0.98157 A^−1^ was obtained. The two ^3^He-CF_4_-filled ORDELA detectors with an active area of more than 1.86 m^2^ and resolution of 5 mm were used to detect the scattered neutrons. We performed corrections for an empty cell and D_2_O. Data were processed by the Mantid software, version 3.13.0, (Mantid—Data analysis and visualization package for neutron scattering and SR experiments), and their analysis was carried out in the SASfit software, version 0.94.10 [43].

### 2.5. Nuclear Magnetic Resonance (NMR)

The ^19^F NMR spectra were measured using a Bruker Avance III HD 400 MHz (Bruker, Billerica, USA). The remaining spectra (^1^H, DOSY, NOESY, HSQC) were acquired on a Bruker Avance III 600 MHz (Bruker, Billerica, MA, USA) NMR spectrometer equipped with a cryogenically cooled probe. The sample temperature was calibrated using a perdeuterated methanol sample [45]. After each change in temperature, the samples were maintained at the given temperature for 15 min prior to the acquisition of spectra. The chemical shift was calibrated using a temperature-dependent water signal according to reference [46].

The *T*_1_ and *T*_2_ relaxation properties of ^19^F atoms were measured with a Varian NMR System 400 MHz (Varian, Palo Alto, CA, USA) equipped with an ID/PFG (pulsed field gradient) probe. The samples were incubated for at least 15 min at the given temperature before the assessment. The *T*_1_ parameter was measured with an inversion recovery experiment (*D*_2_ = 0.2, 0.4, 0.8, 1.6, 3.2, 6.4, 12.8, 25.6, 51.2, 102.4, 204.8, 409.6, 819.2, 1638.4 and 3276.8 ms; 180° pulse width optimized to ≈ 180°; 90° pulse was derived from 180° pulse width). The *T*_2_ parameter was measured with CPMG-sequence (*D*_2_ = 0.05 ms; n = 4, 8, 16, 32, 64, 128, 256, 512, 1024, 2048, 4096, 8192, 16,384, 32,768 and 65,536; 180° pulse width optimized to ≈ 180°; 90° pulse was derived from 180° pulse width). The fitted parameters can be seen in Figures S7 and S8.

For NMR measurements, 2.0 mg of polymers was dissolved in 1.00 mL of H_2_O/D_2_O (volume ratio 9:1) in order to maintain deuterium field-frequency lock and to keep exchangeable amide protons observable. The dominant water signal was suppressed using the excitation sculpting method [47].

The ^1^H spectrum (16 scans, relaxation delay 10.00 s), DOSY (32 scans, size of the FID 2048 by 32, spectral width 12.0 ppm, relaxation delay 2.00 s), multiplicity-edited ^1^H-^13^C HSQC experiment (2 scans, size of the FID 2048 by 400, spectral width 16.0 ppm, relaxation delay 1.50 s) and NOESY spectra (16 scans, size of the FID 2048 by 256, spectral width 10.0 ppm, relaxation delay 2.00 s, mixing time 100 ms) were acquired.

### 2.6. Transmission Electron Microscopy (TEM)

Nanoparticles were measured by TEM using a Tecnai G2 Spirit Twin 12 (FEI Company; Czech Republic) microscope. Sample (polymer concentration 5 mg∙mL^−1^ in 150 mM PBS) and tools for sample preparation (tweezers, microscopic grids, etc.) were placed in an oven heated to 50 °C and left there for 15 min. Afterwards, 2 μL of the sample at 50° was deposited onto a TEM carbon-coated copper grid and left to evaporate in a heated oven at 50 °C, and the dried sample was observed using TEM microscopy at room temperature.

## 3. Results and Discussion

### 3.1. Dynamic Light Scattering (DLS) and Transmission Electron Microscopy (TEM)

The thermoresponsive character of investigated HF polymer was measured by DLS in three different aqueous solutions, 150 mM PBS, H_2_O and D_2_O with a polymer concentration 1 mg∙mL^−1^. The PBS measurements were performed to investigate the characteristics of the HF polymer self-assemblies in an environment with a similar ionic strength to the biological environment, since the ionic strength can have an impact on the thermoresponsive behaviour and the lower critical solution temperature (LCST). The measurements in D_2_O were performed to compare the results collected by SANS, which required measurement in D_2_O, while the results with H_2_O were used in lieu of D_2_O measurements to ensure compatibility with the NMR measurements [48,49,50,51].

**Figure 1:**
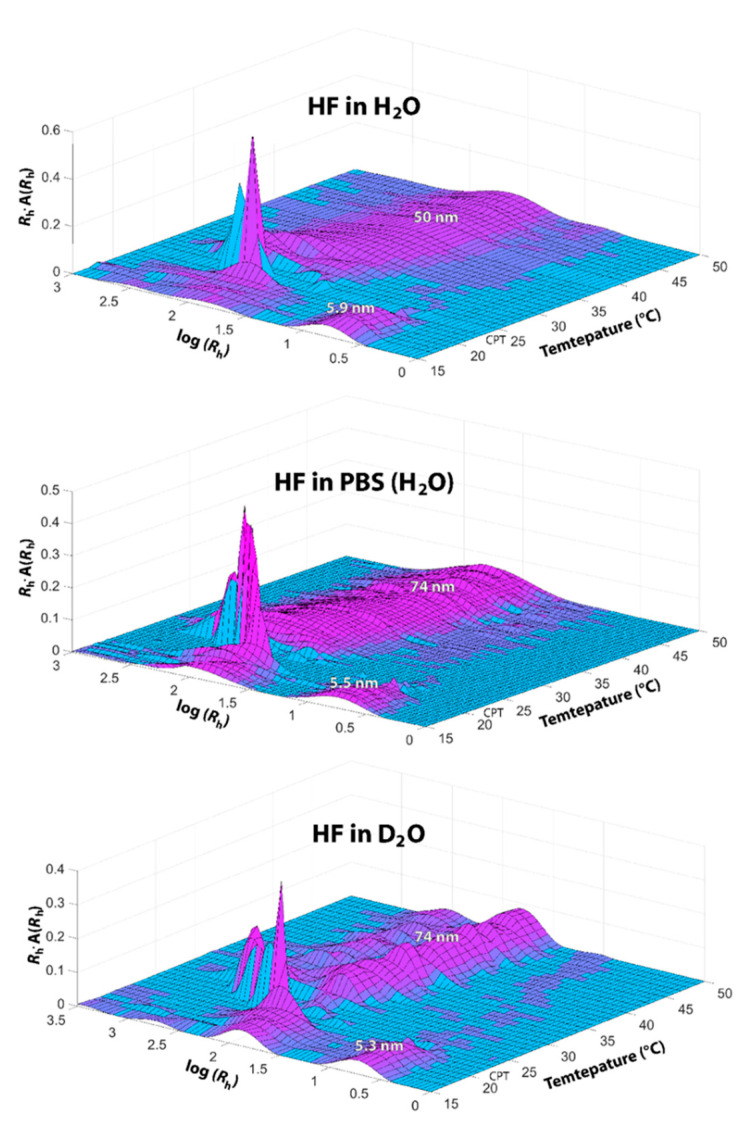
Temperature dependence of self-assembly of HF polymer in PBS, H_2_O and D_2_O measured by DLS. In graphs, we use equal-area representation (the vertical axis shows *R*_h_·A(*R*_h_), where A(*R*_h_) is the intensity distribution function of hydrodynamic radii *R*_h_ of particles in the sample) to maintain legibility.

Based on these measurements, we obtained intensity-weighted distributions of hydrodynamic radii (*R*_h_) and also the cloud point temperatures (CPT), which are the temperatures at which macroscopic phase separation of the investigated polymer occurs at the particular concentration. In general, the polymers at the CPT begin to aggregate into assemblies from the molecularly dissolved polymer chains in the solvent. The polymer assemblies are represented by particle population A (assemblies), and molecularly dissolved polymeric chains are represented by particle population U (unimers). The *R*_h_ values of these particle populations detected during our DLS experiments are listed in Table 1, and their intensity-based distributions are shown in Figure 1. The CPTs, also listed in Table 1, are obtained from DLS results as the temperatures at which the unimer population U disappears and the size distribution changes significantly. Additional depictions of the intensity-based size distributions of particles, which provide a comparative picture of size distributions from Figure 1 below and above the CPT, are presented in Figures S3–S5.

The size difference between unimers in population U and assemblies in population A plays an important role in the circulation time as the unimers can be cleared out from the human body by renal filtration but the assemblies generally have a prolonged circulation time. Another advantage is that particles up to 200 nm in size can enter and concentrate in certain types of solid tumours by the enhanced permeation and retention (EPR) effect.

The thermoresponsive behaviour of our copolymer shows a moderate variation between the solvents used. Obtaining this data is therefore important to conduct a reliable comparison of results from experimental methods carried out in different environments [48,49,50,51].

The transformation from unimers to assemblies is accompanied by so-called anomalous micellization, which is an effect observed in some thermoresponsive copolymer samples [52]. This effect is suspected to be caused by even a trace amount of homopolymer remaining after the synthesis. In DLS size distributions, as shown in Figure 1, the anomalous micellization is visible as a high-intensity peak at the CPT.

The measured particle populations, mainly with regard to the assemblies, have a wide range, which indicates that the present aggregated particles are not uniform in size. To provide some numerical basis of reference for this, we fitted the individual peaks representing the unimers below CPT and assemblies above CPT with a normal distribution model. Normal distributions can generally be described by a probability density function

(2) $$f\left(x\right)=\frac{1}{\sigma \sqrt{2\pi }}e^{-\frac{1}{2}{\left(\frac{x-\mu }{\sigma }\right)}^{2}}$$

where *μ* is the mean of the distribution and *σ* is its standard deviation. The fitting of the measured peaks was carried out on a logarithmic scale because when a linear scale was used, the peaks obtained from the data analysis were asymmetrical. To comprehensibly describe the resulting fitted normal distributions on a linear scale, we present the means *μ* and the outer limits of the central 2*σ*-region (*μ ± σ*) of the distributions in Table 2.

The listed *R*_h_ values in Table 1 are the most prevalent particle sizes of the particle populations shown in Figure 1 and correspond to the means *μ* of the fitted normal distributions. These values were calculated as averages of the positions of maxima of corresponding peaks in a set of neighbouring measurements at the temperature they were measured. These measurements were chosen from regions below or above the CPT where the sample showed good stability and was not affected by an ongoing thermally caused change.

Where the (*μ ± σ*) values describe the width of the particle distribution, the *σ*_Rh_, which are the standard deviations of the peak maxima positions obtained from the calculation of *R*_h_ values, describe the measurement precision and stability of the sample.

In a similar case described in a previous study [12], a polymer containing the poly(2-methyl-2-oxazoline) (PMeOx) formed more than one population of particles above the CPT—one population was described as micelles and the second, larger population, as physically crosslinked nanogels. In this study, our polymer (HF) creates only the larger population of assemblies, which we will further analyse to verify the previously proposed architecture of physically crosslinked nanogels.

**Figure 2:**
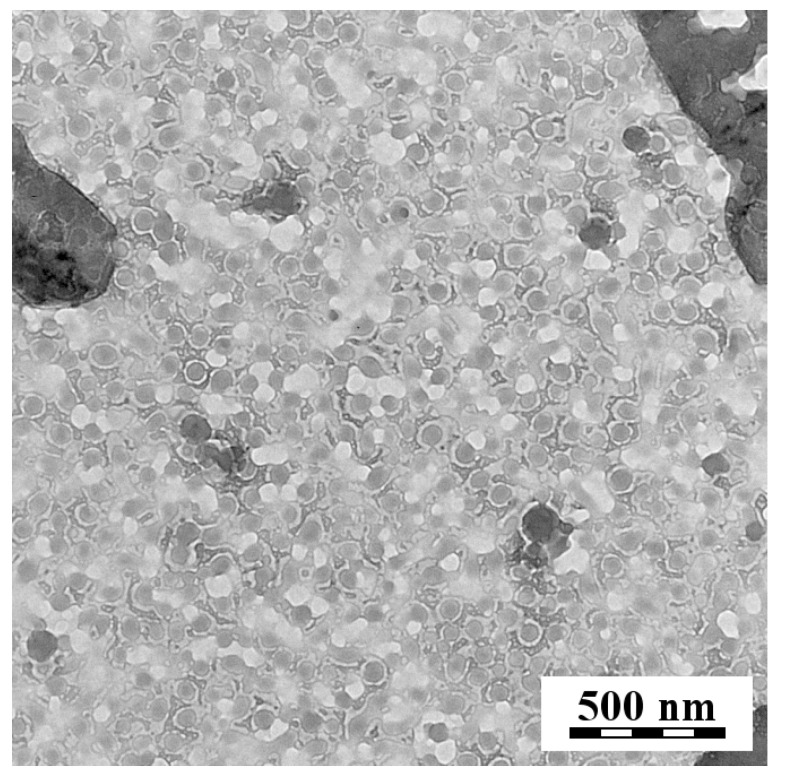
TEM image of dried HF polymer nanoparticles. The scale bar represents 500 nm.

Additionally, we collected the TEM micrograph (Figure 2). The TEM images provide additional information that supports the more detailed and precise DLS results, where we can obtain more valuable information about the particle motion in solution, including its solvation layer. The solvent was evaporated from the sample (polymer concentration 5 mg∙mL^−1^ in 150 mM PBS) at temperatures of approximately 50 °C, and the dried polymer particles were observed using TEM at room temperature. Therefore, the difference between the size distributions obtained from DLS and TEM was caused by the different environments in which the particles were deposited.

### 3.2. Small-Angle X-ray Scattering (SAXS)

**Figure 3:**
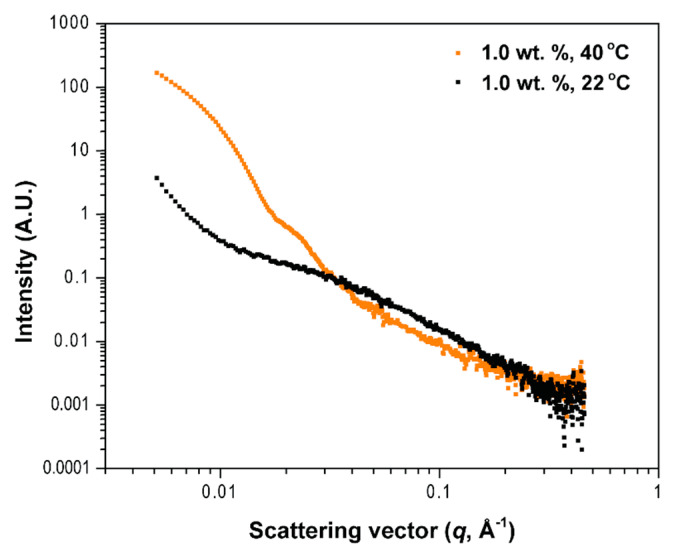
Small-angle X-ray scattering intensities as a function of the scattering vector at different temperatures (22 °C and 40 °C), 1.0 *wt*% polymer solution in PBS.

The samples were further studied by SAXS. As is seen from scattering intensity profiles (Figure 3), the behaviour of the copolymer in PBS buffer at and above the CPT is different to that below the CPT, clearly demonstrating the particle formation process. To obtain parameters of the objects measured in these experiments, we used a superposition of several models (see below).

It is important to note that the lower measurement temperature (22 °C) is similar to the CPT and as such, provides a unique insight into the self-assembly processes of our copolymer. In order to describe data at lower temperature, we had to separate the scattering curve into two contributions. At a lower *q*-range, an increase in intensity can be seen. This correlates with the presence of larger aggregates, as measured by DLS. As a result of the limitations of the experimental technique used, we were able to propose the radius of these aggregates during the self-assembly as being larger than 52 nm. This estimation was obtained using the mass fractal function as a general model. The obtained mass fractal dimension for this model was 2.6, which suggests a dendritic-like or swollen object, which is in good agreement with our findings regarding the swelling degree of aggregates, as discussed below. The data from the higher *q*-range were analysed using the generalized Gaussian coil function. This shows that the radius of gyration of unimers below CPT was 4.65 ± 0.01 nm and that the polymer appears to be in a good solvent—its chains are relaxed and well solvated—as the calculated Flory exponent ν was around 0.52. The Flory exponent indicates whether the polymer is in a good solvent (ν = 0.6) or a poor solvent (ν = 0.33). This result is in good agreement with both the DLS and SANS results.

The particles above CPT were present as loose spheres with a radius of gyration *R*_g_ = 24 nm and internal structure formed by Gaussian chains of *R*_g_ = 1.8 nm.

We calculated the swelling degree Q of particles present above CPT from the measured scattering data using the following formula,

(3) $$Q=\frac{M_{w}\left(V\right)-M_{w}\left(P\right)}{M_{w}\left(V\right)}\cdot 100\%$$

In this equation, *M_w_*(*V*) is the molecular weight calculated from the particle volume and *M_w_*(*P*) is the molecular weight of the swollen particle calculated from the scattering curve. The full calculation can be found in reference [12].

The swelling ratio was calculated as approximately 34% for the sample with a concentration of 1 mg·mL^−1^ using the calculated *M_w_*(*V*) = 6.45∙10^7^ g·mol^−1^ and *M_w_*(*P*) = 4.28∙10^7^ g·mol^−1^. This indicates that a significant amount of water is present in the particles.

### 3.3. Small-Angle Neutron Scattering (SANS)

We analysed the SANS data in a similar manner to the SAXS data. Again, it was necessary to utilize two models simultaneously to obtain a satisfactory fit of the scattering curves. The size results obtained through this analysis were in good agreement with the SAXS experiments, given that these data were acquired in different solvents (PBS for SAXS and D_2_O for SANS) because of the different methods used. However, the DLS results show that at least from the perspective of the hydrodynamic radius, *R*_h_, the samples behave very similarly in all the solvents used.

**Figure 4:**
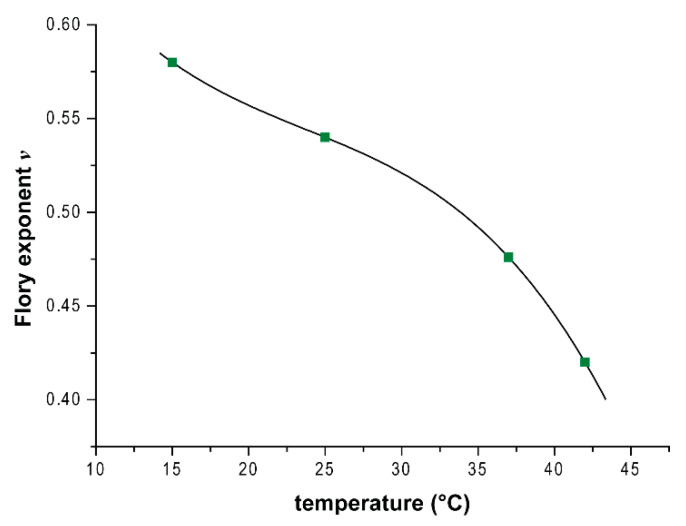
The measured (**green points**) and interpolated (**line**) evolution of the Flory exponent across the experimental temperature range.

From the SANS experiment, we were also able to acquire data on the evolution of the Flory exponent *ν* of the polymer chains with changing temperature. We saw a decrease in the Flory exponent of the HF polymer from 0.58 at 15 °C to 0.42 at 42 °C. This is in accordance with the observation that our samples self-assembled upon heating as the polymer’s hydrophobicity increased. This is represented graphically in Figure 4.

### 3.4. ^1^H and ^19^F NMR Measurements

**Figure 5:**
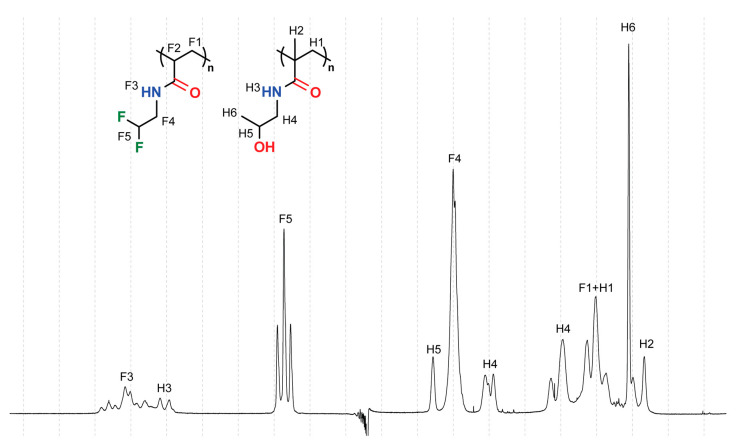
Spectral assignment of ^1^H NMR spectrum (acquired at 25.0 °C) of the HF polymer sample.

**Figure 6:**
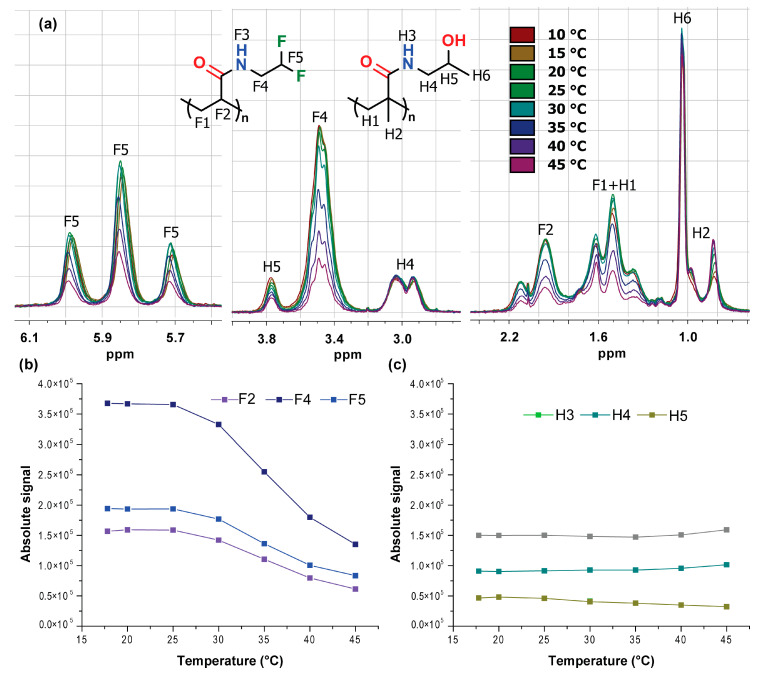
(**a**) Temperature dependence of ^1^H NMR spectra from 10 °C to 45 °C. Regions with important peaks are magnified; (**b**) signal integrals from PDFEA and (**c**) PHPMA as a function of temperature.

Proton signals of PDFEA and PHPMA blocks in HF polymer were assigned as shown in Figure 5 (taking into account the multiplicity-edited ^1^H-^13^C HSQC spectra, Figure S6). Signal integrals in the ^1^H NMR spectrum were monitored as a function of temperature, and the obtained data were presented in Figure 6. Generally, when a polymer collapses and forms particles with low mobility, its NMR signal disappears (due to a shortening of the *T*_2_ time), and only the dissolved, mobile, non-aggregated polymer chains can be observed. Therefore, the observed signal integral of the polymer as a function of temperature can be used to estimate the fraction of the polymer that is collapsed.

In our case, we observed a different behaviour for individual copolymer blocks: the ^1^H NMR signal integrals of the thermo-responsive PDFEA block decay to 40% (compared to integrals at 25 °C; see Figure 6), but the hydrophilic PHPMA remains nearly constant (within the margin of an error, the variations are mainly caused by the overlaps of the signals with PDFEA; see Figure 5). These results are in agreement with expectations, and they suggest that PDFEA partly forms a rigid core of particles above the cloud point temperature and that PHMPA remains very flexible.

**Figure 7:**
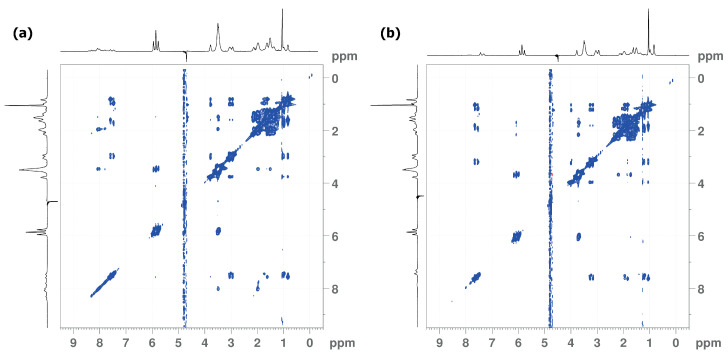
A comparison of NOESY spectra acquired at (**a**) 20 °C and (**b**) 40 °C. The projections of the NOESY spectra were set for the same contour levels; only positive signals were shown to limit the noise after the water signal suppression. External ^1^H projection was used.

The NOESY spectra reveal the spatial proximity of hydrogen atoms that are separated by less than ca. 5 Å and are presented in Figure 7. It is observed that amide protons F3 of the PDFEA block have a spatial proximity to all other protons in this block but do not show cross-peaks with PHPMA in the case of the HF polymer. In the case of HF polymer, significant cross-peaks are observed between F4 and F1/ F2 and between H4/ H5 and H2 (Figure 7). These atoms are located at a large distance in the polymer molecule (or particle). NOESY spectra demonstrate the close contact of sidechains with the backbone. Altogether, these findings are in an agreement with the hydrogen bonding scheme suggested in our previous paper [10]: the blocks are largely segregated and come into only minor contact; since no interaction between different copolymer blocks is observed, sidechains interact exclusively within the block. Note that this interaction is already present in the solvated state of polymers, before the aggregation. The NOESY spectra at higher temperatures are basically identical (except for the disappearance of amide protons at higher temperature) as we observe only the dissolved polymer molecules and not the aggregated ones.

**Figure 8:**
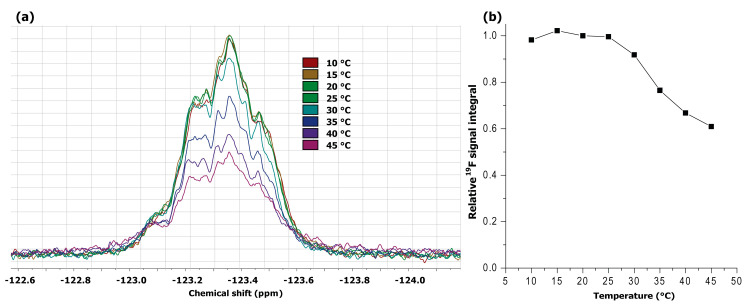
(**a**): Stacked ^19^F NMR spectra of HF polymer at different temperatures; (**b**) the relative integral of the detected ^19^F signal as a variance of temperature.

The studied polymer can potentially be used as an ^19^F MR-imaging tracer, so ^19^F NMR spectra were measured as a function of temperature (Figure 8). The polymer exhibits a single signal peak at −123 ppm. The linewidth (and the non-homogeneous shape of the peak) is determined both by relaxation and spread of chemical shifts resulting from the different proximal structures around the CHF_2_ moiety. The relative detected ^19^F NMR signal decreased when the sample was heated above 20 °C, but the decrease was considerably less pronounced than in ^1^H NMR of the same moiety (see Figure 6), which is fully consistent with the previous reports of similar systems [3,10,12]. In addition to the decreased integral, we observe a minor broadening at the signal “foot”, which suggests another signal component with a short *T*_2_ relaxation corresponding to larger particles. The difference between the temperature dependence of fluorine and proton spectra can be explained by the relaxation properties of ^19^F/ ^1^H atoms in the CHF_2_ moiety. In ^1^H spectra, the aggregated part of the polymer has such a low *T*_2_ that it cannot be easily observed in regular solution NMR spectroscopy. However, the non-aggregated part of the polymer (visible in the NMR) appears unchanged in the spectrum.

The situation is somewhat different in ^19^F NMR. The decrease in the ^19^F signal with increasing temperature in the same moiety is considerably less pronounced than that in ^1^H NMR (see Figure 6 and Figure 8). This indicates that even the polymer aggregates can be observed with a standard ^19^F NMR (in other words, ^19^F NMR may allow one to observe larger polymer aggregates than those observable in ^1^H NMR spectroscopy).

Therefore, ^19^F *T*_1_ and *T*_2_ relaxation times were measured below (20 °C) and above (37 °C, 45 °C) the transition temperatures. The *T*_2_ relaxation times were unchanged by the aggregation within the margin of an experimental error, and the *T*_1_ was slightly prolonged (see Table 3). This result is very promising for the use of the HF polymer as an ^19^F MRI tracer, and it is in full agreement with what was observed in similar systems [11].

Translational self-diffusion coefficients were measured by the pulsed field gradient NMR method. In these DOSY spectra, a decrease in signal intensity for individual ^1^H peaks was monitored as a function of the applied magnetic field gradient strength. Each signal thus provides an independent measure of diffusion properties experienced by the corresponding hydrogen atom. When a molecule moves as a whole (translation of the entire particle), the same diffusion constant should be measured for all its individual ^1^H signals. The observed diffusion coefficients are, therefore, a combination of translational diffusion (its value is essentially the same for both blocks) and the “self-diffusion” caused by deformations of the polymer chain. However, as can be seen in Figure 6, the PHPMA block has noticeably higher diffusion coefficients (although for a portion of the plot, their slopes are similar). This indicates that even before the aggregation, the PDFEA polymers exhibit a lesser degree of freedom (possibly due to higher steric occupation, more rigid bonds between monomers, pre-aggregation particles formation or their more hydrophobic nature compared to PHMPA). PHPMA, on the other hand, exhibits a higher degree of freedom (until the transition temperature).

According to Stokes–Einstein theory, the diffusion coefficient has a linear dependency on temperature and inversely on solvent viscosity, as can be seen in Equation (4).

(4) $$D=\frac{k_{B}T}{6\pi \eta R_{h}}\;$$

**Figure 9:**
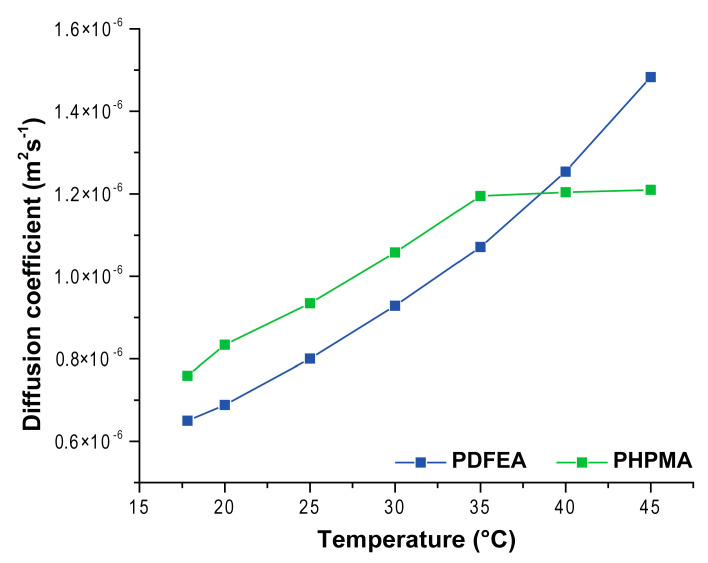
Self-diffusion coefficients determined for HF polymer and its blocks (PDFEA, **blue**, F5; and PHPMA, **green**, H4) as a function of temperature.

The viscosity η decreases with increasing temperature, thereby increasing the diffusion coefficient. Within the range of temperatures used in this study, we can approximate this dependency using a linear function. The linear fit to self-diffusion of water resonance measured in our polymer samples provides a correlation coefficient $R^{2}$ ≥ 0.999 (data not shown). However, diffusion coefficients determined for the polymer signals are not linear within the whole temperature range (see Figure 8), and there is a discrepancy above the transition temperature. We observed that the apparent diffusion coefficient of the PDFEA block has an accelerated increase with temperature. As the temperature increases above the transition point, a portion of PDFEA aggregates (see also Figure 6 and Figure 9). Therefore, the observed PDFEA signal is considerably biased: the observed diffusion coefficients provide information about only the non-aggregated polymer chains, which may be responsible for the apparent continuous increase in the diffusion coefficients. The PHPMA block does not solidify (in accordance with Figure 6); therefore, it shows the unbiased diffusion coefficients of the formed particles. As a result of aggregation, the diffusion becomes limited and becomes nearly constant (slightly increasing with rising temperature).

## 4. Conclusions

The majority of the copolymer PHPMA-*b*-PDFEA is molecularly dissolved below its CPT, with a minor part aggregating into larger particles most likely caused by homopolymers left over from synthesis. The presence of these homopolymers is documented by the observation of anomalous micellization at the CPT. Above the CPT, the copolymer self-assembles into nanoparticles that are too big to be simple micelles.

These particles were confirmed to contain a significant amount of water (approx. 34%), which causes significant swelling of hydrophilic PHPMA chains in these particles and grants them a sufficient freedom of movement so that their ^1^H NMR signals are not diminished by the aggregation process. This is in contrast to ^1^H NMR signals of the PDFEA chains, which decrease significantly. From this, we conclude that the thermoresponsive PDFEA chains, which are hydrophobic above the CPT, form a sort of interconnected network running throughout the aggregated particle. The hydrophilic PHPMA chains then bind the water inside the particle and shield the PDFEA chain, which becomes hydrophobic above the CPT, as documented by the obtained Flory exponents. This assembly can be generally described as a form of hydrated nanogel. It is worth noting that the properties of our sample do not vary significantly in different tested solvents across the investigated range of temperatures, which is a significant difference compared to the PMOX-*b*-PDFEA samples investigated previously. This leads us to the conclusion that the difference in the hydrophilic block has a major impact on the properties of copolymer nanoparticles.

The ^19^F NMR experiments confirmed the ability of our copolymer to provide a detectable ^19^F NMR signal even in an aggregated state above the CPT. This favourable behaviour appears to be connected to the different relaxation properties of the ^19^F nuclei when compared to those of the ^1^H nuclei on the same copolymer block.

This demonstrates that the investigated copolymer could be a viable ^19^F MRI tracer under human physiological conditions and that its nanoparticulate nature could possibly be exploited for the diagnosis of certain tumour types.

Our results, when considered together with previous research, also suggest that more emphasis should be placed on investigating the actual internal structures of copolymer nanoparticles as not all amphiphilic diblock copolymers spontaneously form micelles but can have other architectures as well.

## Figures and Tables

**Table 1:** Size values *R*_h_ and their standard deviations *σ*_Rh_ with regard to neighbouring measurements for unimers (U) and assemblies (A), and cloud point temperatures (CPT) of HF polymer in H_2_O, D_2_O and PBS based on DLS measurements.

Solvent	Unimers	Assemblies	CPT (°C)
	*R*_h_ ± *σ*_Rh_ (nm)	*R*_h_ ± *σ*_Rh_ (nm)	
H_2_O	5.9 ± 0.4	50 ± 6	23
PBS	5.5 ± 0.3	74 ± 7	21
D_2_O	5.3 ± 0.3	74 ± 5	23

**Table 2:** Results of the normal distribution fit of peaks corresponding to unimers (U) and assemblies (A) in H_2_O, PBS and D_2_O describing the width of the individual population peaks with regard to their means *μ*.

Solvent	Unimers			Assemblies		
	*μ* (nm)	*μ − σ* (nm)	*μ + σ* (nm)	*μ* (nm)	*μ − σ* (nm)	*μ + σ* (nm)
H_2_O	6.0	4.0	9.1	52	24	117
PBS (H_2_O)	5.7	3.5	9.1	75	34	165
D_2_O	5.8	3.6	9.3	83	41	165

**Table 3:** *T*_1_ and *T*_2_ relaxation parameters determined at 9.4 T magnetic field; presented as values ± fid-based SD. Fitting curves can be seen in Figure S7 and S8; all were fitted with $R^{2}$ ≥ 0.998.

*t* (°C)	*T*_1_ (ms)	*T*_2_ (ms)
20.0	363 ± 66	142 ± 9
37.0	452 ± 9	127 ± 8
45.0	462 ± 14	178 ± 9

## Supplementary Materials

The following are available online at https://www.mdpi.com/2079-4991/10/11/2231/s1: Figure S1. Size exclusion chromatography (SEC) trace for HF polymer (concentration 5 mg mL^−1^ polymer solution in mobile phase). SEC was measured by HPLC Ultimate 3000 system (ThermoFisher Scientific, Dionex, Waltham, Massachusetts, USA) equipped with an SEC column (TSKgel SuperAW3000, 150 ∙ 6 mm, 2.4 μm), three detectors (UV−vis detector, refractive index Optilab-rEX detector and multi-angle light scattering DAWN EOS, Wyatt Technology Co., Santa Barbara, California, USA, detector) and with a methanol and sodium acetate buffer (0.3 M, pH 6.5) mixture (80:20 *wt*%, flow rate of 0.5 mL∙min^−1^) as the mobile phase. Figure S2. ^1^H NMR spectra were measured using Bruker Avance III 600 MHz (Bruker, Billerica, USA) NMR spectrometer (16 scans, relaxation delay 10.00 s, 2.0 mg of polymers were dissolved in 1.00 mL of methanol-d4). Figure S3. Intensity-based size distributions of HF polymer in H_2_O depicted using equal area-representation below (blue data) and above (black data) the CPT. Figure S4. Intensity-based size distributions of HF copolymer in PBS (H_2_O) depicted using equal-area representation below (blue data) and above (black data) the CPT. Figure S5. Intensity-based size distributions of HF copolymer in D_2_O depicted using equal-area representation below (blue data) and above (black data) the CPT. Figure S6. HSQC-edit MF polymer (2 mg in 1 mL of H_2_O/D_2_O 9:1), 2 scans, size of the FID 2048 by 400, spectral width 16.0 ppm, relaxation delay 1.50 s. Figure S7. *T*_1_ relaxation of ^19^F nuclei determined by inversion recovery experiment in 9.4 T field. The sample was dissolved in H_2_O/D_2_O (90:10), and the experiment was conducted at 20.0, 37.0 and 45.0 °C. Figure S8. *T*_2_ relaxation of ^19^F nuclei determined by Carr–Purcell–Meiboom–Gill sequence in 9.4 T field. The sample was dissolved in H_2_O/D_2_O (90:10), and the experiment was conducted at 20.0, 37.0 and 45.0 °C.
