# Supplementary material for: Internal Structure of Thermoresponsive Physically Crosslinked Nanogel of Poly[N-(2-hydroxypropyl)methacrylamide]-Block-Poly[N-(2,2-difluoroethyl)acrylamide], Prominent 19F MRI Tracer
Source: Nanomaterials (Basel). 2020 Nov 10;10(11):2231. doi: 10.3390/nano10112231 (PMC7698257; doi:10.3390/nano10112231)
Supplement: Supplementary file 1 [file nanomaterials-10-02231-s001.pdf]

Supporting Information for article

Internal Stucture of Thermoresponsive Physically Crosslinked Nanogel of poly[*N*-(2-hydroxypropyl)methacrylamide]-*block*-poly[*N*-(2,2-difluoroethyl)acrylamide], Prominent ^{19}F MRI Tracer

David Babuka,[1,2#] Kristyna Kolouchova,[1,3#] Ondrej Groborz,[1,4,5] Zdenek Tosner,[6] Alexander Zhigunov,[1] Petr Stepanek,[1] Martin Hruby,[1]*

[1]Institute of Macromolecular Chemistry, Czech Academy of Sciences, Heyrovského náměstí 2, 162 06 Prague 6, Czech Republic

[2]Institute of Physics, Faculty of Mathematics and Physics, Charles University in Prague, Ke Karlovu 3, 121 16 Prague 2, Czech Republic

[3]Department of Physical and Macromolecular Chemistry, Faculty of Science, Charles University, Hlavova 8, 128 43 Prague 2, Czech Republic

[4]Department of Organic Chemistry, Faculty of Science, Charles University, Hlavova 8, 128 43 Prague 2, Czech Republic

[5]Institute of Biophysics and Informatics, Charles University, First Faculty of Medicine, Salmovská 1, 120 00 Prague 2, Czech Republic

[6]Department of NMR spectroscopy, Faculty of Science, Charles University, Hlavova 8, 128 43 Prague 2, Czech Republic

*Corresponding author, E-mail: mhruby@centrum.cz

[#] Both authors contributed equally.

Keywords: Fluorine, nuclear magnetic resonance, self-assembly, diblock, nanoparticle architecture, PHPMA, PDFEA

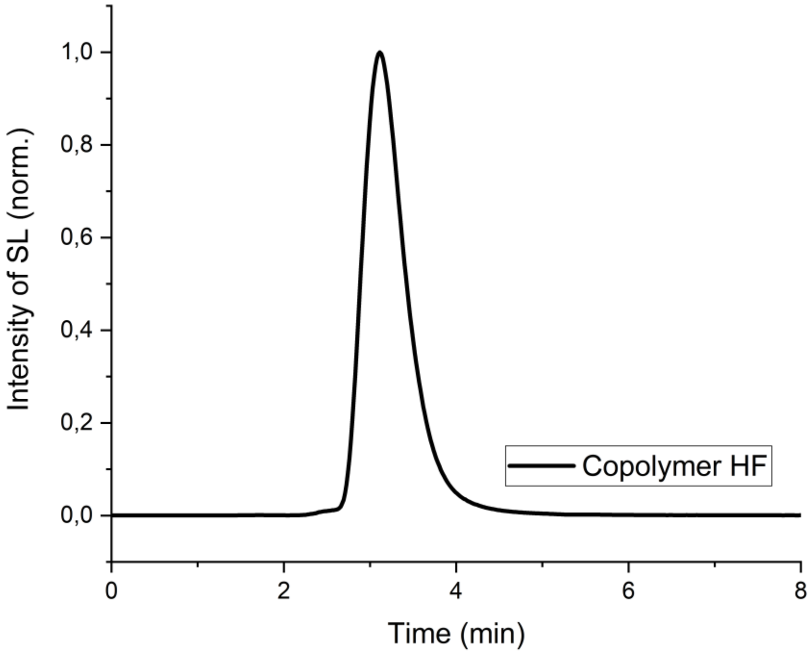

Figure S1. Size exclusion chromatography (SEC) trace for polymer HF (concentration 5 mg mL^{-1} polymer solution in mobile phase). SEC was measured by HPLC Ultimate 3000 system (ThermoFisher Scientific, Dionex, USA, Waltham, Massachusetts) equipped with an SEC column (TSKgel SuperAW3000, 150×6 mm, 2.4 µm), three detectors (UV−vis detector, refractive index Optilab-rEX detector, and multi angle light scattering DAWN EOS, Wyatt Technology Co., USA, detector) and with a methanol and sodium acetate buffer (0.3 M, pH 6.5) mixture (80:20 w %,flow rate of 0.5 mL min^{-1}) as the mobile phase.

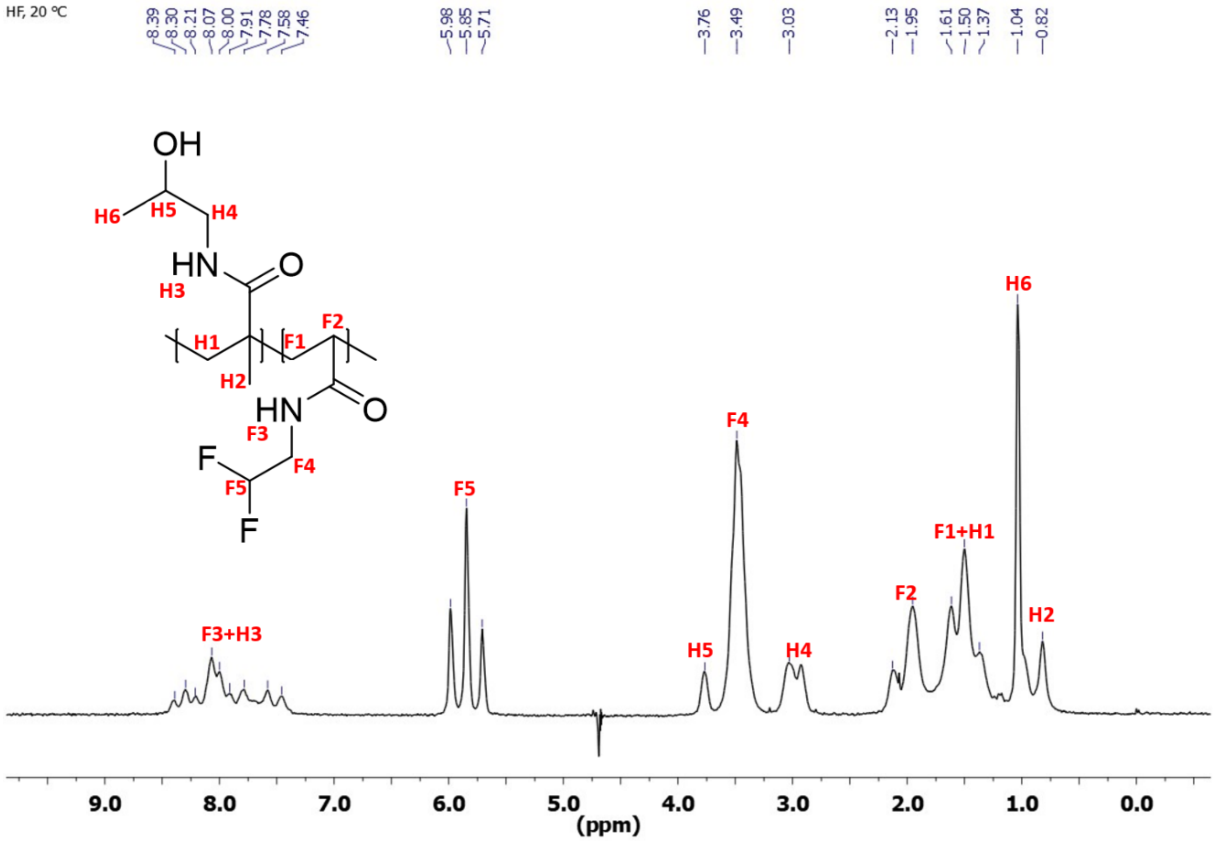

Figure S2. ^{1}H NMR spectra were measured using Bruker Avance III 600 MHz (Bruker, Billerica, USA) NMR spectrometer (16 scans, relaxation delay 10.00 s, 2.0 mg of polymers were dissolved in 1.00 mL of *methanol*-d4).

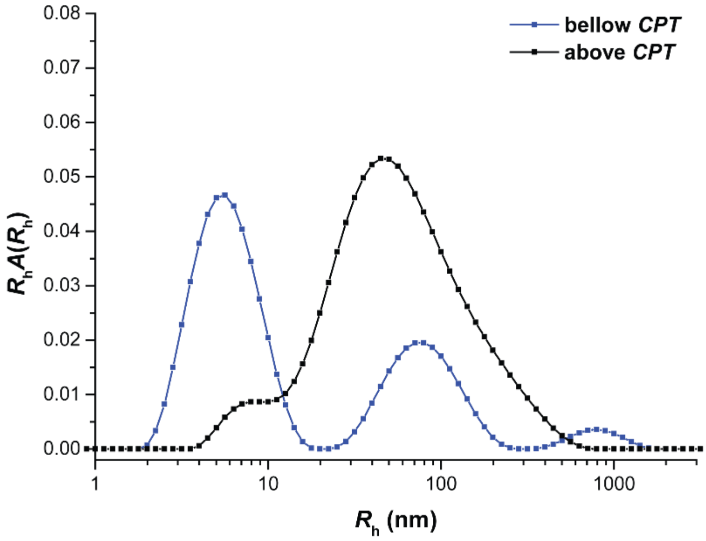

Figure S3. Intensity based size distributions of HF copolymer in H_2O depicted using equal-area representation below (blue data) and above (black data) the CPT.

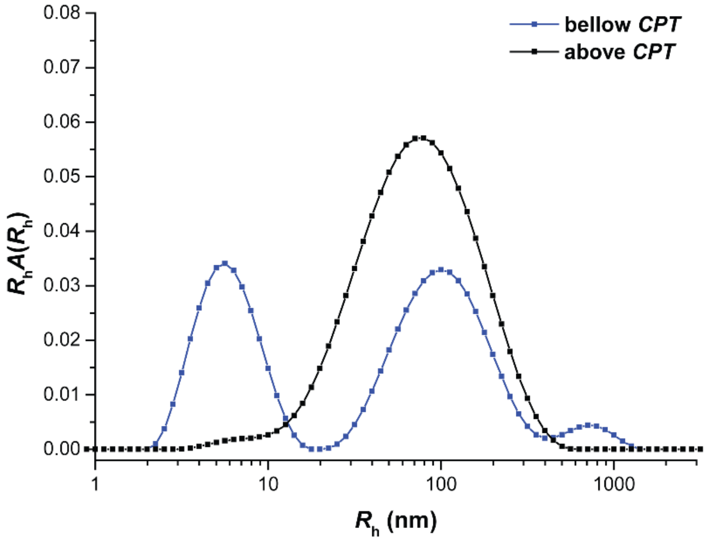

Figure S4. Intensity based size distributions of HF copolymer in PBS (H_2O) depicted using equal-area representation below (blue data) and above (black data) the CPT.

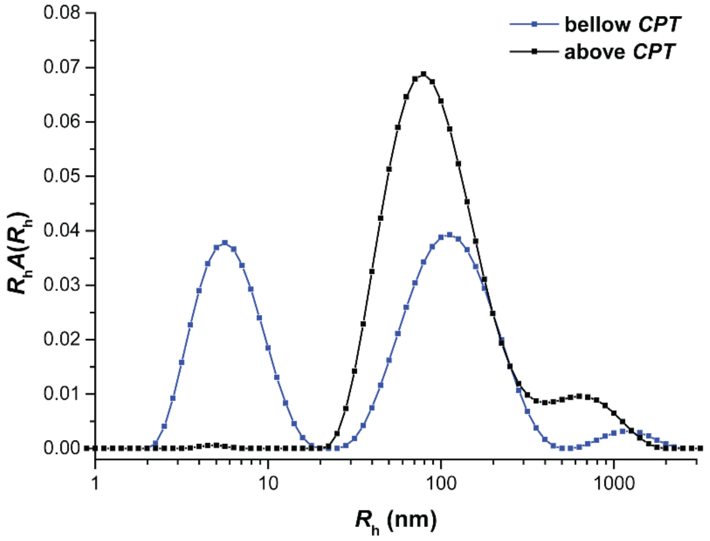

Figure S5. Intensity based size distributions of HF copolymer in D_2O depicted using equal-area representation below (blue data) and above (black data) the CPT.

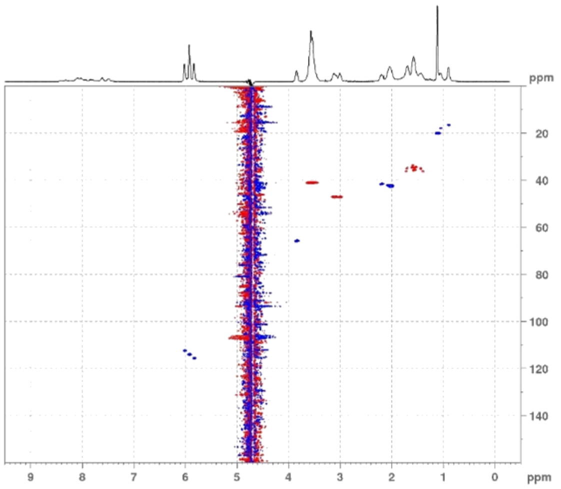

Figure S6. HSQC-edit MF polymer (2 mg in 1 mL of H_2O/D_2O 9:1), 2 scans, size of the FID 2048 by 400, spectral width 16.0 ppm, relaxation delay 1.50 s.

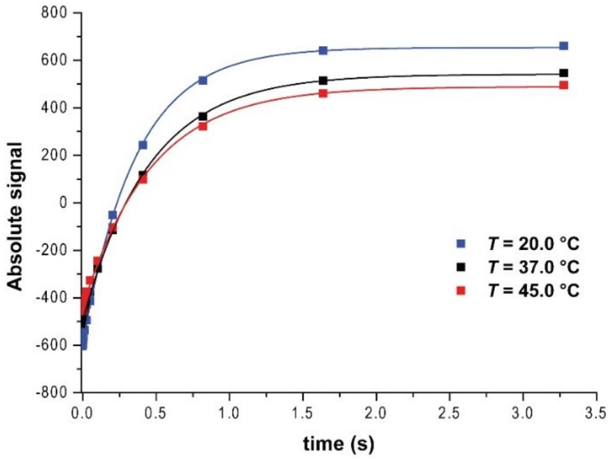

Figure S7. T_1 relaxation of ^{19}F nuclei determined by inversion recovery experiment in 9.4 T field. The sample was dissolved in H_2O/D_2O (90:10) and the experiment was conducted at 20.0, 37.0 and 45.0 °C.

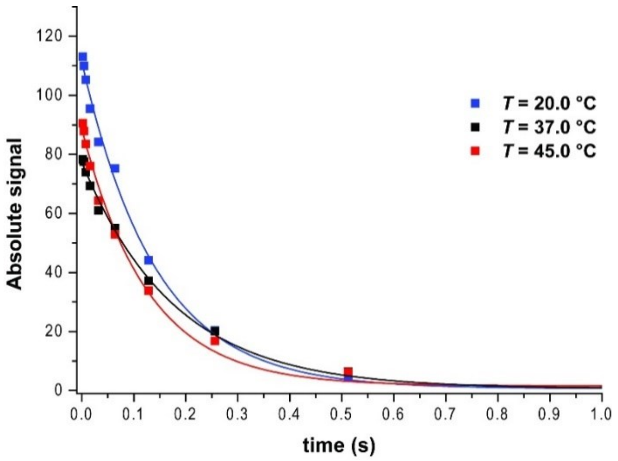

Figure S8. T_2 relaxation of ^{19}F nuclei determined by Carr-Purcell-Meiboom-Gill sequence in 9.4 T field. The sample was dissolved in H_2O/D_2O (90:10) and the experiment was conducted at 20.0, 37.0 and 45.0 °C.
